# Corrigendum

**DOI:** 10.1002/elsc.202170033

**Published:** 2021-05-03

Unfortunately, in the article

**Characterization and potential antitumor effect of a heteropolysaccharide produced by the red alga *Porphyridium sordidum***

By Biliana Nikolova, Severina Semkova, Iana Tsoneva, Georgi Antov, Juliana Ivanova, Ivanina Vasileva, Proletina Kardaleva, Ivanka Stoineva, Nelly Christova, Lilyana Nacheva and Lyudmila Kabaivanova,

https://doi.org/10.1002/elsc.201900019,

the second affiliation of the author Georgi Antov was erroneously omitted. The correct affiliations for Prof. Dr. Antov are:

^1^ Institute of Biophysics and Biomedical Engineering, Bulgarian Academy of Sciences, Sofia, Bulgaria

^2^ Institute of Plant Physiology and Genetics, Bulgarian Academy of Sciences, Sofia, Bulgaria

